# Cardiovascular Collapse Secondary to Beta-Blocker Administration in a Setting of Coexisting Thyroid Storm and Atrial Fibrillation: A Case Report

**DOI:** 10.7759/cureus.29321

**Published:** 2022-09-19

**Authors:** Syed Faqeer Hussain Bokhari, Huma Sattar, Shaun Abid, Rimsha R Vohra, Samar Sajid

**Affiliations:** 1 Medicine and Surgery, Mayo Hospital, Lahore, PAK; 2 Internal Medicine, Sharif Medical and Dental College, Lahore, PAK; 3 Internal Medicine, Allama Iqbal Medical College, Lahore, PAK; 4 Internal Medicine, Dow University of Health Sciences, Karachi, PAK; 5 Medicine, Dow University of Health Sciences, Karachi, PAK

**Keywords:** cardiovascular collapse, cardiac arrest, diltiazem, beta-blockers, thyroid storm, atrial fibrillation

## Abstract

A thyroid storm is a rare endocrinological emergency caused by severe hyperthyroidism. Reducing circulating levels of free T3 in blood and beta-adrenergic inhibition are the basis of medical treatment for thyroid storms. Propranolol, due to its additional effect of preventing the peripheral conversion of dormant T4 to active form T3, is the chosen drug for blockade in hyperthyroidism and thyroid storm. We describe a rare clinical case of cardiovascular collapse following propranolol administration in a setting of thyroid storm. The patient presented with symptoms of dyspnea and palpitations and had an ejection fraction of 10%. He was started on a calcium channel blocker (diltiazem). Further investigations revealed that the patient also had a thyroid storm and was immediately shifted to methimazole and propranolol. However, following the administration of a beta-blocker, the patient developed circulatory failure as a result of cardiac arrest, necessitating the use of vasopressors and inotropes. This implores the need for further investigations and treatment regimens for cardiovascular conditions, especially atrial fibrillation arising in thyrotoxicosis, as there are no solid treatment guides in the literature to the best of our knowledge.

## Introduction

Thyroid storm (TS) is an uncommon entity with a reported incidence of 16% of thyrotoxic hospitalized patients in the United States survey [1]. It is a life-threatening condition characterized by multiorgan dysfunction caused due to excessive thyroid hormone production. TS has severe cardiovascular manifestations ranging from tachycardia, hypotension, and congestive heart failure to cardiac arrhythmias and death from cardiovascular collapse. Even though initial management is well known in patients with a history of longstanding untreated hyperthyroidism and includes beta-blocker, thionamides, iodine solution, and glucocorticoids, yet early recognition and management of TS are challenging in patients with no known thyroid disease and TS as an initial presentation [2,3]. Propranolol, a non-cardio-selective blocker, is an anti-adrenergic drug that also has the effect of preventing the peripheral conversion of inactive thyroxine (T4) to the active thyroid hormone triiodothyronine (T3) [4]. Patients with thyroid crisis may have clinical or subclinical thyrotoxic cardiomyopathy, which predisposes them to an excessive reaction to a beta-blocker medication, resulting in cardiac collapse [1]. We present a case of TS in a young male patient with no reported past medical history of hyperthyroidism who presented with a complaint of palpitations and developed cardiac arrest following propranolol administration. Before the cardiovascular collapse, he initially received Cardizem for noted atrial fibrillation and subsequently beta-blockers after his lab work-up showed findings concerning thyrotoxicosis. All this happened because of the use of beta-blocker in hyperthyroidism without knowing about thyrotoxic cardiomyopathy. So far, the literature is lacking in defining an algorithm to guide treatment in this kind of situation and therefore requires further studies to be done.

## Case presentation

**Figure 1:**
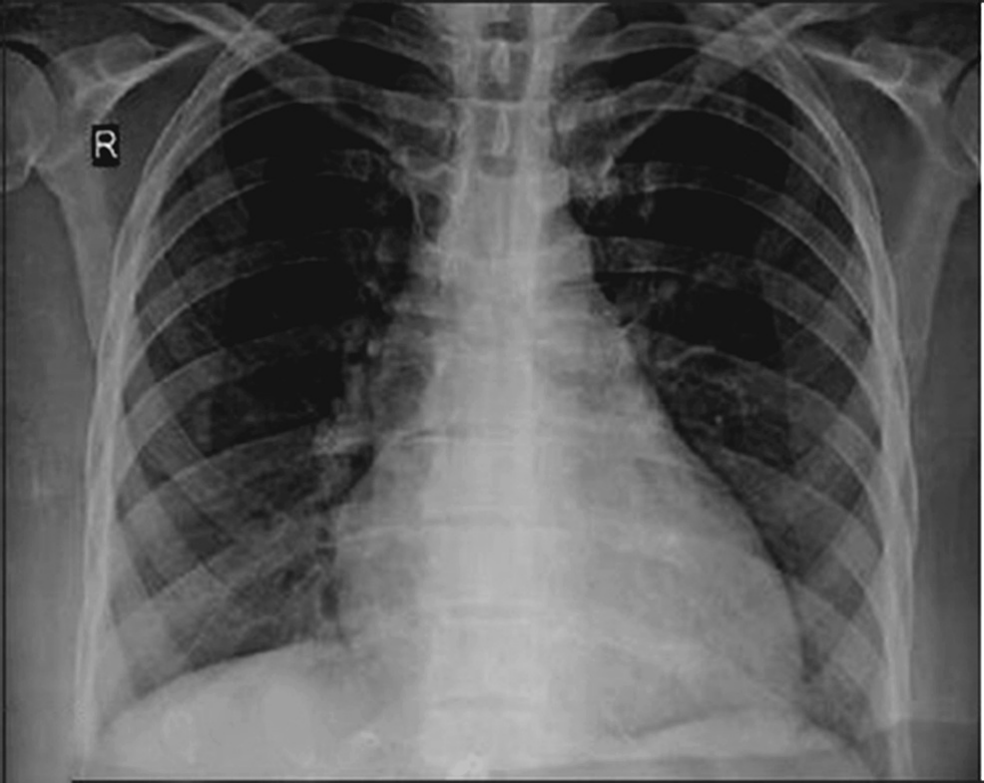
X-ray of the chest showing severe cardiomegaly.

A 57-year-old male with a past medical history of generalized anxiety disorder presented to the ED with a complaint of dyspnea and palpitations. Initial examination showed a heart rate of 193 bpm and a temperature of 37.80 C. ECG revealed atrial fibrillation. Initially, he was administered Cardizem (diltiazem), but no change in heart rate was observed. Laboratory examination revealed significantly low thyroid stimulating hormone (TSH) of 0.005 and free T4 of 3.87, indicating thyrotoxicosis. Furthermore, increased free T3 levels were also observed (>20). Diltiazem drip was immediately stopped, and the case was discussed with the consultants of the endocrinology department. Burch-Wartofsky Point Scale (BWPS) for thyrotoxicosis score was calculated, which turned out to be greater than 45, indicating a TS. The patient was started on methimazole and propranolol. Atrial fibrillation was persistent but was rate controlled. Warfarin was started for anticoagulation. Within six hours after the administration of methimazole and propranolol, the patient became hypotensive, severely bradycardic, and went into cardiac arrest. After achieving a return of spontaneous circulation via cardiopulmonary resuscitation (CPR) and defibrillation, a glucagon drip was administered to reverse the effect of propranolol. Following an attempt with external transcutaneous pacing, a transvenous pacer was inserted per electrophysiologist recommendations. The patient was started on vasopressors and was admitted to the ICU. X-ray of the chest revealed severe cardiomegaly (Figure 1). CT scan of the chest showed bilateral pleural effusions and dilated pulmonary artery, indicating pulmonary artery hypertension. A very low ejection fraction of 10% was found on an echocardiogram. An intra-aortic balloon pump was placed, which was successfully removed a few days later. The patient also tested positive for COVID-19 infection; however, neither remdesivir nor a monoclonal antibody was given since COVID-19 was not thought to be the likely cause of his respiratory symptoms. After that, a second echocardiography revealed an ejection fraction of 30%. The patient was downgraded to the telemetry unit since his hemodynamics improved, and his T3 and T4 levels trended down to 3.90 and 0.98, respectively.

The patient again developed atrial fibrillation in the telemetry unit, and IV digoxin pushes were administered. Warfarin was continued, and amiodarone was also started on a scheduled basis for anticoagulation and rhythm control. He then subsequently shifted to a sinus rhythm followed by sinus bradycardia. Additionally, he experienced encephalopathy while in the hospital. MRI and CT scans of the brain did not identify any apparent abnormalities. Cultures of blood and urine revealed no bacterial growth. Severe pharyngeal dysphagia diagnosed during the videofluoroscopic swallow study (VFSS) was documented by speech therapy. The patient received antibiotic treatment for potential aspiration pneumonia. The patient's mentation eventually returned to baseline during the hospital stay. Due to occasional bradycardia throughout his hospital stay, beta-blockers were not prescribed. He was discharged on losartan and aspirin for his cardiomyopathy and methimazole for thyrotoxicosis. Moreover, he was deemed not a candidate for a life vest on discharge because of his significant deconditioning, and rather close cardiology follow-up on out-patient was advised. 

## Discussion

Hyperthyroidism has a variety of effects on the cardiovascular system [5]. These effects are achieved by the hormone's direct influence on extranuclear cell components or by stimulating thyroid nuclear receptors, leading to gene transcription [6]. This results in a high cardiac output (CO) condition due to the combined effects of greater contractility, better diastolic relaxation, and lower peripheral resistance. This helps hyperthyroid individuals to adjust for the abnormally high metabolic requirement by increasing CO levels in the resting state. During stressful situations, such as exercise, the body fails to raise CO enough to satisfy the superimposed exercise-induced increase in metabolic demand, a condition known as a decreased contractile reserve [7]. The hyperthyroid-related loss in the contractile reserve has been linked to the symptoms of heart failure (HF) in the presence of high CO [8]. This is frequently referred to as "high-output" HF. However, many have argued that the phrase is misleading [9] since CO levels stay higher during both rest and stress phases, and decompensation is functional, meaning it is not linked to the failure or discontinuation of the high output state [10]. Furthermore, although rare, persistent severe hyperthyroidism can cause HF with low CO levels. Low-output failure is most often multifactorial in hyperthyroid individuals [11].

Thyrotoxic crisis, also known as TS, is a severe type of hyperthyroidism and a rare endocrinological emergency with a 10%-30% fatality risk [12]. The pathophysiology is complicated, with an exaggerated response to T3 and a sudden rise in free hormone levels due to a reduction in protein carrier capacity. It causes a hyperadrenergic condition in which the body's sensitivity to circulating catecholamines is increased. The hormone's ability to raise -adrenergic receptor density by amplifying production and reducing degradation achieves this [13]. Medical, surgical, and supportive care are all used in the treatment [3]. The mainstays of medical treatment include lowering blood T3 levels and blocking the hormone's peripheral effects via -adrenergic blockade [13]. Non-cardio-selective blockers (NCBB) have been widely employed as the mainstay of treatment in both thyrotoxic crisis and simple hyperthyroidism, in an attempt to prevent this hyperadrenergic condition [14]. Propranolol has been a popular NCBB since it has the added benefit of preventing the peripheral conversion of dormant T4 to active T3 [12].

Propranolol may cause an unusual but significant adverse reaction in persons with pre-existing clinical or preclinical thyrotoxic cardiomyopathy. NCBB administration may prevent the compensatory mechanism caused by the thyrotoxicosis-induced hyperadrenergic condition, resulting in a considerable drop in cardiac output in the context of stress, such as a thyroid crisis, leading to circulatory collapse [4]. When our patient came with atrial fibrillation, he did not have indications of pre-existing thyrotoxic cardiomyopathy. Shortly after administering propranolol, he experienced significant hypotension, followed by cardiac arrest. The diagnosis of propranolol-induced cardiogenic shock was supported by echocardiographic observations of low ejection fraction and poor cardiac contractility due to the temporal link between propranolol administration and the abrupt onset of hemodynamic instability. In the literature, 11 cases of beta-blocker-induced circulatory collapse in thyroid crisis have been reported [1]. Almost all patients exhibited signs of thyrotoxic cardiomyopathy previous to the adverse event, with five having a confirmed poor ejection fraction. Six of them died of cardiac arrest, while the remainder died of hypotension. The most often prescribed beta-blocker was propranolol. The presence of thyrotoxic cardiomyopathy, particularly in low-output heart failure, can lead to an excessive reaction to a beta-blocker medication, resulting in circulatory collapse and cardiogenic shock.

## Conclusions

Administration of a beta-blocker, such as propranolol, in a setting of TS, may result in thyrotoxic cardiomyopathy. This worsens the patient's hemodynamic condition, resulting in severe hypotension, bradycardia, and cardiac arrest. The medical literature has reported no proper therapeutic regimen for this condition. A detailed analysis of such cardiovascular complications arising in thyrotoxicosis is required, and more studies are needed to formulate a concrete therapeutic regimen for such conditions. This may help to reduce the mortality associated with such cases.
